# Gene-environment interaction in ADHD traits: the role of school environment, personality, callousness-unemotional traits and satisfaction with life

**DOI:** 10.1007/s00787-024-02628-y

**Published:** 2025-01-06

**Authors:** Inga Schwabe, Dirk H. M. Pelt, Corina U. Greven

**Affiliations:** 1 Department of Methodology and Statistics, Tilburg University, PO Box 90153, Tilburg, 5000 LE The Netherlands; 2 Department of Biological Psychology, Vrije Universiteit Amsterdam, Amsterdam, The Netherlands; 3 Amsterdam Public Health Research Institute, Amsterdam University Medical Center, Amsterdam, The Netherlands; 4 Department of Cognitive Neuroscience, Radboud University Medical Centre, Nijmegen, The Netherlands; 5 Donders Institute for Brain, Cognition and Behaviour, Radboud University Medical Center, Nijmegen, The Netherlands; 6 Karakter Child and Adolescent Psychiatry University Centre, Nijmegen, The Netherlands

**Keywords:** Gene-environment interaction, ADHD, IRT, Hyperactivity-impulsivity, Inattentiveness

## Abstract

We investigated gene-environment interaction in ADHD traits, focusing on environmental variables related to *personality traits*, *school environmen*t, *satisfaction with life*, and *callousness*. Using data from 2170 16-year-old twins and state-of-the-art methodology to prevent spurious findings due to measurement scale artifacts, gene-environment interaction models were estimated separately for the two core ADHD dimensions, *hyperactivity-impulsivity*, and *inattentiveness*. Based on scores on the SWAN questionnaire, where high scores are reflective of low ADHD traits, results showed that additive genetic influences were more important in explaining individual differences in the degree of *hyperactivity-impulsivity* in twins with a high score on the trait *conscientiousness* than in twins with a moderate or low score (e.g., a *positive* gene-environment interaction). Similarly, additive genetic influences were relatively more important in explaining individual differences in the degree of *inattentiveness* in twins with a high score on *conscientiousness* or *satisfaction with oneself*. Lastly, a *negative* gene-environment interaction was found: For twins with a high score on the trait *callousness*, additive genetic influences were less important in explaining differences in the degree of *inattentiveness* than in twins with an average or low score on the trait. The finding of these specific gene-environment interactions is important for a deeper understanding of the etiology of ADHD traits and may assist in developing targeted interventions for genetically vulnerable individuals.

**Supplementary Information:**

The online version contains supplementary material available at 10.1007/s00787-024-02628-y.

## Introduction

While genetic influences are strongly implicated in the etiology of attention-deficit hyperactivity disorder (ADHD) (heritability estimates ranging from 70 to 90%, see Nikolas & Burt [1] for a meta-analysis), it remains difficult to characterize the *specific* environmental circumstances under which ADHD symptoms emerge. The reason for this difficulty might lie in the complex nature of ADHD traits (i.e., *hyperactivity-impulsivity* and *inattentiveness*): Emerging evidence suggests that complex traits are associated with interactions between genetic- and environmental influences. In the behavior genetics literature, this phenomenon is referred to as *gene-environment interaction* and in case of ADHD traits, this means that a genetic predisposition for an ADHD trait might determine the relative importance of environmental influences in explaining individual differences in ADHD traits. Yet, research on gene-environment interaction has not been a prominent focus of earlier research: Although many studies have been conducted, most of them investigated an interaction between environments and *specific* genes (so-called *candidate gene studies* – see for example Ansari et al. [2]. However, it has been shown that results of candidate gene studies do not reliably replicate [3]; a phenomenon that has been attributed to various factors among which insufficient sample sizes and the highly polygenic nature of ADHD (i.e., many genes of individually small effects) [4]. More recently, studies started utilizing *polygenic risk scores* which summarize this polygenicity in a single score representing an individual’s genetic susceptibility to an ADHD trait. For example, Plomin et al. [5] explored gene-environment interaction in the prediction of teacher rating of behavior problems (among which ADHD traits) from 6687 twins at ages 7,9 and 10 from parent reports of children’s environment at ages 3 and 4. They found significant but modest (the largest effect accounting for 0.4% of the variance) interactions with no clear developmental trends across childhood. The largest effect suggested that children with a high genetic susceptibility for ADHD traits (i.e., high polygenic risk scores) showed more conduct problems when their parents were high in discipline. Mooney et al. [6] used a cross-sectional design to study interactions between environmental factors^1^ and among others ADHD polygenic risk scores in two independent data sets (*N* = 11,876; *N* = 1449). Although multiple gene-environment interactions were observed, they were confounded by ancestry and did not replicate. Similar to Plomin et al. [5], the authors conclude that interaction effects are likely small for polygenic risk scores and the examined environmental variables. Another study design that can be used to investigate the relative role of genes and environments is the *classical twin design*, which compares the similarity in a trait among identical (monozygotic, MZ) and non-identical (dizygotic, DZ) twins (cf [7]). This design makes use of the fact that DZ twins share on average 50% of their segregating genes while MZ twins are genetically identical: These known genetic relationships make it possible to estimate the proportion of the total variance in a trait (referred to as *phenotypic variance*) that can be explained by three different sources of variation (parametrized as latent variables in a structural equation model): additive genetic influences (A) that capture the combined genetic effect of all genes, common-environmental influences (C) that capture any factors perfectly shared within a twin pair (e.g., environmental influences experienced in a shared home or school) and unique-environmental influences (E) that capture all environmental influences not shared between twins (e.g., personal experiences or friends) including measurement error. An extension of the classic twin design allows the investigation of gene-environment interaction, where the effect of environmental influences is dependent on genetic influences in explaining variation in a trait. For example, this could mean that for twins with a high genetic predisposition for ADHD, environmental influences are more important in creating individual differences regarding hyperactivity-impulsivity symptoms than for twins without such a predisposition.

In a recent study, item-level data of 2168 16-year-old twin pairs were used to investigate gene-environment interaction separately for the two ADHD dimensions *hyperactivity-impulsivity* and *inattentiveness* [8]. To investigate gene-environment interaction, the study used an omnibus test, which means that, instead of focusing on the effect of one (or more) *measured* environmental stressors in the twin design (like for example a measurement of chaos at home or the school environment), gene-environment interaction was parametrized as an interaction between the latent variables. Focusing on *unique-*environmental influences only, a (latent) interaction was modelled between the latent variables that represent additive genetic influences (A) and unique-environmental influences (E) in the twin model. This parameterization results in proper statistical power to determine–in an exploratory way- whether there is *any* gene-environment interaction at all. Results indicated that the relative importance of unique-environmental influences in explaining differences in both ADHD dimensions was dependent on a twin’s degree of genetic predisposition for the two dimensions (e.g., a *positive* gene-environment interaction).

Although this finding contributes to our understanding of the etiology of ADHD, a drawback of the used method is that we do not know what *specific* unique-environmental measures are important in explaining this gene-environment interaction.

Here, we extend previous research by focusing on the following question: can we identify interactions between additive genetic influences relevant for ADHD traits and *specific* measured environmental measures? Although various studies suggest that gene-environment interaction is an important phenomenon in mental disorders (see e.g [9])., research on interactions between additive genetic influences and specific environmental stressors has not been a prominent focus in ADHD research. Based on a sample of 520 twin pairs, Gould et al. [10] focused on two environmental factors: socioeconomic status (SES) and chaos (household disorganization). They fitted four different gene-environment models, separately for ADHD traits (hyperactivity-impulsivity and inattentiveness) and the two environmental variables of interest (SES and chaos). Results suggested that neither chaos nor SES were associated with a change in the extent to which additive genetic influences explain variability in ADHD traits. These findings seem to argue against the presence of gene-environment interaction, at least in the population studied. The difference between our study and the study by Gould et al. [10] is two-fold: (1) we focus on an extended and systematically selected set of environmental variables, and (2) we apply a psychometric approach that corrects for increased measurement error in the tails of ADHD scores. The latter is important since the application of traditionally used methods can result in the spurious finding of a gene-environment interaction effect: The amount of information obtained from a questionnaire typically varies for respondents suffering from different degrees of ADHD. That is, if both affected and healthy individuals are assessed with a scale that also contains extreme items (like for example “*I always have a hard time to pay attention*”), then the resulting scale scores may be very reliable for twins that show a high degree of ADHD symptoms but can be very unreliable for healthy twins. In other words: Measurement error is not homogeneous among all degrees of ADHD. As a result, the distribution of composite scores (like sum-scores) is skewed. Research has shown that this skewness can result in the finding of *spurious* gene-environment interaction effects [11–13]. Instead, here, we used a statistical method that uses item-level data by simultaneously estimating the genetic twin model and an item response theory (IRT) measurement model. As such, the phenotypic variance decomposition (including the estimation of gene-environment interactions) is done directly on the latent variable (corrected for measurement error) rather than on composite scores. This prevents the finding of a spurious interaction effect, making results artifact-free (see [14] for technical details).

We assessed gene-environment interactions in 2170 16-year-old twins who completed the *Strength and Weaknesses of ADHD Symptoms and Normal Behavior* (SWAN) questionnaire as well as a broad range of measured environmental variables. Based on the earlier finding of an interaction between additive genetic influences and *unique*-environmental influences [8], we concentrated on environmental variables that differ within twin pairs (i.e., influences not shared within a twin pair).

## Method

We preregistered all of our hypotheses and analyses (see https://osf.io/5bkw8/).

### Data

Data originated from the *Twins Early Development Study* (TEDS) [15], a longitudinal study that recruited twin pairs born between 1994 and 1996 in England and Wales (for more information, see https://www.teds.ac.uk/). For this study, we used the data of twins with available item-level data on the SWAN questionnaire, which assesses ADHD as a continuous trait. From this sample, a total of *N* = 379 individual twins were excluded (*N* = 59 due to medical reasons, *N* = 189 due to being perinatal outliers, *N* = 34 due to a lack of initial contact, *N* = 66 due to a lack of zygosity information, *N* = 5 due to both medical reasons and being perinatal outliers and *N* = 26 due to both a lack of initial contact and lack of information on zygosity). This resulted in a total sample of twins with SWAN item information of 2170 individual twins (1085 twin pairs) of which 419 were monozygotic (MZ) twin pairs and 666 dizygotic (DZ) twin pairs. Of the MZ twin pairs, 140 twin pairs were male and 279 female. Of the DZ twin pairs, 128 were male, 216 pairs were female and 322 were opposite-sex twin pairs. Mean age of the twins at the time of returning the questionnaire was 16 years and three months (SD = 0.75, minimum age = 14 years and 9 months, and maximum age = 18 years and 7 months).

### Selection of environmental variables

We considered all environmental variables that were collected by TEDS in the sample of 16-year-old twins that can be regarded as measures of unique-environmental influences and were assessed via self-report (i.e., questionnaires answered by twins themselves). In this paper, we adopt a broad definition of what variables are considered environmental variables, also including personality traits like callousness or conscientiousness. It is important to note that some of the environmental variables might be phenotypically related to ADHD traits and/or contribute to present gene-environment correlations (see the Discussion for more detail), but this was corrected for in the statistical model by including the main effects of environmental variables in the means part of the model and as such regressing out any covariance between environmental variables and ADHD. Consequently, gene-environment effects are also estimated after controlling for the presence of gene-environment correlations (see the paragraph on pleiotropy below for more detail but also Pelt et al. [16] and van der Sluis et al. [17]). To systematically select variables for the gene-environment interaction analyses, a literature review and psychometric analyses were conducted (see the online supplementary material). Below, you can find a detailed description of the selected environmental variables (for all considered variables see the preregistration). For all selected environmental variables, we calculated factor scores for every individual twin and used them as a proxy in the estimated genetic models.

### Measures

#### ADHD traits (hyperactivity-impulsivity and inattentiveness)

ADHD traits were assessed as self-report (i.e., questionnaire filled in by twins themselves) with the *Strength and Weaknesses of ADHD symptoms and Normal Behavior* (SWAN) questionnaire, developed by Swanson et al. [18]. The rationale for using self-report data here was based on psychometric considerations – SWAN scores are informative at all levels of the two ADHD traits [19] and subscales are reasonably long (e.g., 9 items per subscale) which makes scale scores generally more reliable compared to other scales that were available like the SDQ [8, 19].

The SWAN questionnaire consists of two subscales of 9 items, derived from the DSM-IV-TR [20]. The two subscales measure the two ADHD traits inattentiveness and hyperactivity-impulsivity using both a seven-point scale of behavior. ADHD traits scores were scaled such that a *higher* score is reflective of a *lower* degree of hyperactivity-impulsivity and inattentiveness respectively. Based on Cronbach’s alpha, the reliability of the subscales was respectively 0.79 (inattentiveness) and 0.80 (hyperactivity-impulsivity).

On the hyperactivity-impulsivity subscale, 95% of the twin families had no missing data at all, 2% missed a single item answer within one family, 1% missed a total of 8 or fewer item scores within one family and 2% missed a total of 9 item scores within one family. On the inattentiveness subscale, 94% of the twin families had no missing data at all, 3% missed a single item answer within one family, 1% missed a total of 8 or fewer item scores within one family, and 2% missed a total of 9 or 10 (*N* = 1 individual family) item scores within one family.

All analyses that we conducted were estimated separately for the two ADHD traits hyperactivity-impulsivity and inattentiveness.

#### Personality traits

Personality traits were assessed with the *Five Factor Model Rating Form* (FFMRF [21]) which consists of a total of 30 items representing each of the 30 facets from the *NEO Personality Inventory—Revised* (NEO PI-R [22]). The 30 items are organized with respect to the 5 Big Five dimensions (6 items measuring one dimension) *neuroticism*, *agreeableness*, *openness to experiences*, *conscientiousness*, and *extraversion*. Each item is rated on a 5-point scale ranging from “*1: extremely low*” to “*5: extremely high*”.

#### Callous-unemotional traits

Callous-unemotional traits were assessed by the *Inventory of Callous-Unemotional Traits* (ICU), which consists of a total of 24 items assessed on a 4-point scale, ranging from “*0: Not at all true*” to “*3: Definitely true*” [23]. In the TEDS assessment wave at age 16 years, the youth self-report version of this scale was used. The scale consists of three different subscales: *callousness* (e.g., missing empathy and remorse), *uncaring* (e.g., an uncaring attitude about the performance on tasks and/or other’s feelings), and *unemotional* (e.g., deficient emotional affect).

#### Life satisfaction

Life satisfaction was measured using the *Multidimensional Students’ Life Satisfaction Scale* (MSLSS), which originally consisted of 40 items assessed on a 4-point scale. Based on a pilot study in TEDS, a version of the MSLSS was used that consists of 21 of the original items, assessed on a 4-point scale. These items measured the following five domains: *family* (4 items), *friends* (5 items), *school* (4 items), *living environment* (4 items), and *self* (4 items).

#### School-related measures

Two different measures were used (*classroom environment* and *academic self-concept*). *Classroom environment* was assessed using a combination of items originating from two different scales: 9 of the originally 12 items long *Student classroom environment scale* [24] and 9 items from the *Programme for International Student Assessment* (PISA) classroom environment scale. Academic self-concept was measured using a shortened version consisting of 10 items of the 20-item scale developed by Burde [25].

### Genetic models

The most commonly used model is the ACE model which decomposes the total observed variance in a trait ($\:{\sigma\:}_{P}^{2}$) into variance that can be explained by additive genetic (A) influences ($\:{\sigma\:}_{A}^{2}$), common-environmental (C) influences ($\:{\sigma\:}_{C}^{2}$) and unique-environmental (E) influences ($\:{\sigma\:}_{E}^{2}$) [7]:

$$\:{\sigma\:}_{P}^{2}=\:{\sigma\:}_{A}^{2}\:+\:{\sigma\:}_{C}^{2}\:+\:{\sigma\:}_{E}^{2}$$

Narrow-sense heritability, *h*^*2*^ is then defined as the relative proportion of the total variance that can be explained by $\:{\sigma\:}_{A}^{2}$:

$$\:{h}^{2}=\:\frac{{\sigma\:}_{A}^{2}\:}{{\sigma\:}_{P}^{2}}=\frac{{\sigma\:}_{A}^{2}\:}{{\sigma\:}_{A}^{2}\:+\:{\sigma\:}_{C}^{2}\:+\:{\sigma\:}_{E}^{2}}$$

It is also possible to fit an AE model (fixing the C component to zero) and an ADE model in which common-environmental influences are replaced by dominant genetic influences. Here, we used an AE model for the estimation of gene-environment interaction in hyperactivity-impulsivity and an ACE model for the estimation of gene-environment interaction in inattentiveness, based on an earlier analysis of a subset of the data [8].

#### Modeling gene-environment interaction

An interaction can be parametrized by specifying one (or more, but for readability, the following example will be based on one variable only) environmental variable to modify the log-transformed variance of additive genetic influences [14, 26]. We used this parametrization rather than the traditionally used parametrization by Purcell [27] since it has been shown that the Purcell parametrization is not uniquely identified [14].

Modifying the log-transformed variance of additive genetic influences means that, for twin *j* from family *i*, the additive genetic component is divided into an intercept (estimating additive genetic variance when $\:{\mathbf{E}}_{ij}=0$) and a part that is dependent on the environmental variable (representing the gene-environment interaction):

$${\sigma ^2_{Aij}} = exp({\beta _{0A}} + {\beta _{1A}}{\bf{E}_{ij}})$$

where $\:\mathbf{E}$ denotes a vector consisting of the values of the environmental variable for every twin *j* from family $\:i$ (e.g. academic self-concept), *β*_0A_ represents the intercept (estimating additive genetic variance when $\:{\mathbf{E}}_{ij}=0$) and *β*_1A_ represents the interaction effect (e.g., the interaction between additive genetic influences and for example the environmental measure *academic self-concept*). Using this parametrization, the interaction effect is modeled as a (log)linear effect, meaning that the additive genetic variance component is larger at either higher or lower values of the environmental variable (e.g., larger individual differences) where the direction of the effect depends on the sign of the slope *β*_1A_.

#### Estimating main environmental effects

Van der Sluis et al. [17] showed that a spurious interaction effect can be expected in the case that the environmental variable (e.g., *classroom environment*) and the trait (e.g., *hyperactivity-impulsivity*) are correlated and the values of the environmental variable (e.g., *classroom environment*) are correlated within one twin family as well. They showed that this potential bias can be prevented by correcting the trait value of an individual twin (e.g., *hyperactivity-impulsivity*) for his or her own environmental variable value (e.g., *classroom environment*) but also for the value of the co-twin (e.g., *co-twin’s score on classroom environment*). Furthermore, the values of these regression values should be allowed to differ across zygosity by estimating regression parameters *separately* for MZ and DZ twins [17].

Following the advice by van der Sluis et al. [17], next to including environmental variables in the part of the genetic model that estimates the interaction effect, we also regressed the value of an environmental variable, $\:{\mathbf{E}}_{ij}$ for twin *j* from family *i*, on his/her ADHD traits (e.g., a twin’s latent level of *hyperactivity* or *inattention*, respectively). This resulted in four different estimates for every environmental variable: an estimate of the effect of the environmental variable on the ADHD trait (for example the degree of *hyperactivity*) of the twin self (e.g.,$\:{\beta\:}_{m}$) and of his/her co-twin (e.g., $\:{\beta\:}_{mc}$). Following van der Sluis et al. [17], these two parameters were estimated separately for MZ twin families and DZ families (resulting in a total of four different estimates per environmental variable).

#### Note on pleiotropy between ADHD traits and environmental variables

Note that some of the used environmental variables might also have genetic effects (e.g., not be considered purely environmental variables) and that the underlying genes may *also* be associated with ADHD traits (e.g., there is shared genetic variation, a phenomenon referred to as *pleiotropy* in genetics). For example, a study by Verhoef et al. [28] has shown that some of the genetic variation related to educational attainment is shared across ADHD traits. Given the phenotypic correlation between educational attainment and *classroom environment* (an environmental variable used here), it is likely to expect pleiotropy between ADHD traits and classroom environment as well. However, although it is plausible to expect shared genetic effects, in the model that is used here, the main effect of an environmental variable (like for example *classroom environment)* was incorporated in all gene-environment interaction models by including the main effect in the means part of the model. More specifically, following the advice by van der Sluis et al. [17], we regressed a twin’s value (as well as the value of the co-twin) on the used environmental variables out of the twin’s latent ADHD trait scores. Consequently, any covariance between an environmental variable and latent ADHD traits is regressed out, including genetic covariance^2^ (see also Pelt et al. [16]). Note, however, that this also mean that we cannot distinguish between the different sources of covariances (i.e., genetic or environmental covariance) between ADHD traits and environmental variables nor can we quantify the *strength* of gene-environment correlations (see the Discussion for more detail). Although models exist that make it possible to quantify the source of covariance and/or the strength of gene-environment correlations (see for example [27]), these models are computationally too demanding when IRT models are used to correct for the spurious finding of gene-environment interactions (see also Pelt et al. [16]).

#### Integrated item response theory model

In every model, we integrated an IRT model into the analysis and estimated parameters of both the genetic and the IRT model at the same time (i.e., in one *unified* model). Within the IRT approach, a twin’s latent trait (e.g., level of inattentiveness) is estimated using not only his or her item answers, but also item properties are taken into account, such as the discriminating value of an item. Details of the IRT model that was used for this particular application can be found in the online supplementary material.

### Estimation and selection of the final gene-environment interaction model

To simultaneously estimate the IRT and the genetic model (for technical details, see e.g [11, 29, 30]), we adopted a Bayesian approach. Details can be found in the online supplementary material.

To arrive at a final model, we estimated gene-environment interaction models separately for every category of environmental variables (e.g., 1) *personality traits*, 2) *callous*-*unemotional traits*, 3) *life satisfaction* measures and 3) *school*-related measures). In every model, also the main effect of the respective measures was included, following the procedure described above. This was done separately for both ADHD dimensions, resulting in a total of eight estimated models. Based on the results of these models, a final selection of environmental variables was done, separately for hyperactivity-impulsivity and inattentiveness based on the 95% highest posterior density (HPD [31]) interval (Bayesian analogue of a confidence interval in frequentist statistics) of the gene-environment interactions in the separate models. When the 95% HPD interval did not contain zero (meaning the interaction was significant), the environmental variable was included in the final model.

## Results

Below, we only report the results of the final genetic gene-environment model. Results for all estimated models can be found in the online supplementary material.

### Hyperactivity-impulsivity

The final gene-environment model was an AE model that included main and interaction effects of the environmental variables *academic self-concept*, *classroom environment*, and *conscientiousness*. Point estimates and 95% HPD interval of all relevant parameters can be found in Table 1.

**Table 1:** *Hyperactivity-impulsivity*: estimated parameter values. HPD = highest posterior density interval. *A* represents additive genetic influences. Note that total phenotypic variance was equal to 0.40 (not restricted to be equal to 1)

Parameter	Point estimate	95% HPD
*β*_0A_	0.17	[0.12;0.23]
*β*_0E_	0.23	[0.18;0.28]
*A x academic self-concept*	0.15	[0.02;0.28]
*A x classroom environment*	0.24	[0.06;0.39]
*A x conscientiousness*	0.22	[0.10;0.33]

Narrow-sense heritability of hyperactivity-impulsivity was equal to 0.43. Results suggest that the largest part of the variance in the latent trait *hyperactivity-impulsivity* could be explained by unique-environmental influences, but a substantial percentage of the variance was explained by additive genetic influences as well. Furthermore, there was a positive and significant interaction effect between additive genetic influences and *conscientiousness*. This means that additive genetic influences were more important in explaining individual differences in hyperactivity-impulsivity in twins that score high on conscientiousness than in creating individual differences in hyperactivity-impulsivity in twins with a low or average score on conscientiousness.

As described above, to avoid bias in the estimation of interaction effects, a twin’s hyperactivity-impulsivity scores were corrected not only for his or her value on an environmental variable but also for his or her co-twin’s value, and these regression parameters were allowed to differ across zygosity. Estimates were found to be very similar across zygosity and within the same family (see online supplementary material).

### Inattentiveness

The final gene-environment model for *inattentiveness* was an ACE model including main and interaction effects of the environmental variables *satisfaction with school*, *satisfaction with oneself*, *callousness*, *conscientiousness*, and *academic self-concept*. Point estimates and 95% HPD interval of all relevant parameters can be found in Table 2.

**Table 2:** *Inattentiveness*: estimated parameter values. HPD = highest posterior density interval. Note that total phenotypic variance was equal to 0.54 (not restricted to be equal to 1)

Parameter	Point estimate	95% HPD
*β*_0A_	0.09	[0.03;0.16]
*β*_0C_	0.07	[0.01;0.13]
*β*_0E_	0.38	[0.29;0.47]
*A x Satisfaction with school*	0.24	[-0.02;0.49]
*A x Satisfaction with oneself*	0.34	[0.10;0.56]
*A x Callous*	-0.58	[-0.99;-0.24]
*A x Conscientiousness*	0.40	[0.21;0.58]
*A x Academic self-concept*	0.27	[0.05;0.48]

Surprisingly, results suggest that unique-environmental influences could explain the largest part of the variance in the latent trait inattentiveness. Out of the remaining variance, equal parts could be explained by additive genetic influences and shared environmental influences, resulting in an estimate of narrow-sense heritability equal to 0.16. Furthermore, there were positive and significant interaction effects between additive genetic influences and *satisfaction with oneself* as well as *conscientiousness*. This means that additive genetic influences are more important in explaining individual differences in inattentiveness in twins that score high on satisfaction with oneself than in creating individual differences in inattentiveness in twins with a low or average score on satisfaction with oneself. Likewise, differences in inattentiveness between twins with a high score on the trait conscientiousness are to a higher degree explained by additive genetic influences than are differences in inattentiveness between twins with a moderate or low score on the trait conscientiousness. Lastly, we found a negative and significant (the 95% HPD not including zero) interaction effect between additive genetic influences and callousness, meaning that additive genetic influences are less important in explaining individual differences in inattentiveness in twins that score high on the trait callousness compared to twins that score moderate or low on the trait.

Again, estimates for the main effects of environmental variables were found to be very similar across zygosity and within the same family (see online supplementary material).

## Discussion

Using the item-level data of 2170 16-year-old twins, we estimated gene-environment models separately for the ADHD traits hyperactivity-impulsivity and inattentiveness while focusing on environmental variables related to *personality traits*, *school environment*, *satisfaction with life*, and *callousness*.

### Heritability and variance decomposition

With respect to *hyperactivity-impulsivity*, results in terms of variance decomposition and narrow-sense heritability were similar to those found earlier in a similar subset of the TEDS data [8] although narrow-sense heritability found in this study was lower (e.g., an estimate of 0.43 compared to 0.57 found earlier). This difference might be explained by the fact that *specific* environmental measures were not included before (neither in the estimate of gene-environment interactions nor as main effects). Concerning inattentiveness, the estimated narrow-sense heritability estimate was lower than estimated before (e.g., an estimate of 0.16 compared to 0.24) which again might be explained by the fact that no measured environmental variables were included in the genetic model.

### Gene-environment interactions

For both ADHD traits, we found a *positive* interaction with the trait conscientiousness: Additive genetic influences associated with *hyperactivity-impulsivity* were more important in explaining individual differences in the degree of *hyperactivity-impulsivity* in twins with a high score on conscientiousness than in twins with a moderate or low score. Vice versa, additive genetic influences associated with *inattentiveness* were more important in explaining individual differences in the degree of *inattentiveness* in twins with a high score on conscientiousness compared to those with an average or low score. In other words, with high scores on the SWAN being reflective of a low degree of ADHD, this means that a lower genetic susceptibility towards ADHD traits was found for twins with a high degree of conscientiousness. Another positive interaction was found between additive genetic influences associated with *inattentiveness* and the trait *satisfaction with oneself*: Twins with a high score on satisfaction with oneself showed a lower genetic susceptibility towards a high degree of inattentiveness than those with a low or average score. Lastly, a *negative* gene-environment interaction was found: For twins with a high score on the trait *callousness*, additive genetic influences associated with *inattentiveness* were less important in explaining differences in the degree of *inattentiveness* than in twins with an average or low score on the trait. In other words: A low score on callousness was associated with a lower genetic susceptibility towards a high degree of inattentiveness.

The finding of these specific gene-environment interactions is important for a deeper understanding of the aetiology of ADHD, but can also help clinical practice determine the focus of (preventive) therapy. In a nutshell, results suggest that (1) a *high* score on *conscientiousness* is correlated with a *low* genetic susceptibility for *either one of the ADHD traits*, (2) a *high* score on *satisfaction with oneself* is correlated with a *low* genetic susceptibility for *inattentiveness* and lastly (3) a *low* score on *callousness* is correlated with a *low* genetic susceptibility for *inattentiveness*. In general, results seem to suggest that the direction of gene-environment interactions follow earlier found phenotypic correlations: Concerning the trait conscientiousness, earlier research already found a strong link between conscientiousness and ADHD [32–34]. Our findings suggest that this link might even be stronger given that a genetic susceptibility for a low degree of ADHD traits is associated with high conscientiousness. Likewise, the link between a high degree of ADHD and *callousness* that has been found before [35–37] might be more amplified in adolescents with a genetic predisposition towards a high degree of *inattentiveness*. However, although earlier research has shown that there is a link between ADHD and *satisfaction with life* [38, 39], to our knowledge no research has yet linked ADHD scores specifically to individual differences in *satisfaction with oneself*. This might mean that our finding of an interaction between satisfaction with oneself and additive genetic influences important for the degree of inattentiveness might not necessarily generalized to other populations than the one studied here.

### Strengths and limitations

While most twin studies that focused on ADHD traits used the classic twin design (assuming the absence of interplay between genetic- and environmental influences), the present study explicitly modelled gene-environment interactions. Another strength is that this was done in a very systematic way. Before fitting any genetic models, we (1) executed a literature review, and (2) assessed the psychometric properties of the scales used to measure environmental variables. Another important strength is that we used novel methodology to prevent the finding of a spurious interaction effect due to heterogeneous measurement error.

### Gene-environment correlation

It is important to note that, next to gene-environment interaction, gene-environment correlation (rGE) can be at play. rGE refers to the situation that genetic susceptibility for a trait and environmental circumstances are correlated (i.e., a higher frequency of genetic factors associated with a trait in an environment). In the case of ADHD, for example, adolescents with a high susceptibility for (a high score on) ADHD traits may evoke chaotic environments. The underlying case for an increased heritability of ADHD at higher levels of an environmental variable (i.e., in this example, a more chaotic home) would, in this example, be explained by the presence of rGE rather than a positive gene-environment interaction (example adapted from Gould et al. [10]). However, in all gene-environment models applied here, a twin’s value (as well as the value of his/her co-twin) on the used environmental variables was regressed out of the twin’s latent ADHD trait score. Consequently, any covariance between an environmental variable and latent ADHD traits is regressed out, meaning that we tested gene-environment interactions *after* gene-environment correlation is accounted for.

Although this means that the found interactions are likely to be genuine interaction effects (i.e., not explained by a correlation between genetic and environmental effects), an obvious limitation is that this approach prevents us from quantifying the *strength* of a possible gene-environment correlation. Although models exist that make it possible to estimate gene-environment interactions and correlations *at the same time* (see for example Purcell et al. [27]), these models are known to suffer from methodological problems [40] and would need a much larger sample size and are computationally too demanding in the case that IRT models are used to correct for the spurious finding of gene-environment interactions [16]. We deemed preventing the finding of a spurious interaction more important than *also* quantifying the strength of possible gene-environment correlations but note that a larger sample size that can accommodate this power issue is crucial for future research in this area.

### Assessment of ADHD traits

Furthermore, the assessment of ADHD was based on self-report (i.e., SWAN items answered by twins themselves). However, research has shown that self-reports among children and adolescents typically underestimate the presence of ADHD symptoms as well as result in lower heritability estimates compared to parent- or teacher-based reports. To validate the results of this study, future research should apply multidimensional measurement models (see [41] for an example) that integrate measurements of different instruments based on self-report as well as parent- or teacher reports. Note that this is possible with the data used in this article since the TEDS dataset al.so includes a parent-related measure of ADHD traits (i.e., the Conners scale [42]) but it was out of the scope to integrate both measurement instruments in the used GxE models. Lastly, our findings may not necessarily generalize to clinically diagnosed ADHD adolescents.

## Electronic Supplementary Material

Below is the link to the electronic supplementary material.

Supplementary Material 1

## Data Availability

Data originated from the Twins Early Development Study (TEDS), a longitudinal study that recruited twin pairs born between 1994 and 1996 in England and Wales (for more information, see https://www.teds.ac.uk/).

## Footnotes

1 The included environmental variables consisted of *family income*, *family conflict/negative sentiment*, *geocoded measures of area deprivation*, *lead exposure risk*, and *air pollution exposure*.

2 Note that this also means that gene-environment interactions are tested after gene-environment correlation is accounted for (see Discussion for more detail).
