# Supplementary material for: Gene-environment interaction in ADHD traits: the role of school environment, personality, callousness-unemotional traits and satisfaction with life
Source: Eur Child Adolesc Psychiatry. 2025 Jan 6;34(7):2205–14. doi: 10.1007/s00787-024-02628-y (PMC12334439; doi:10.1007/s00787-024-02628-y)
Supplement: Supplementary file 1 — Supplementary Material 1 [file 787_2024_2628_MOESM1_ESM.docx]

**Online supplementary material**

1. **Selection of environmental variables**

A literature review was performed to identify those variables with the strongest link to ADHD. That is, separately for every potential environmental factor, search terms relating to ADHD and the respective environmental variable were used to search databases, and titles and abstracts were then screened to determine inclusion. Selecting only those articles that reported standardized coefficients, we used reported correlation and/or regression coefficient to quantify the strength of the relationship between ADHD trait and the particular variable, where we used a threshold of |0.30|. The selection of environmental variables to include in gene-environment analyses was further narrowed by evaluating the quality of the measurement where we considered 1) available data (e.g., missingness in scale scores) and 2) the psychometric quality of the scale used to measure the environmental variables. To judge the psychometric quality of the available scales, item response theory (IRT) models were performed and item-total correlations, as well as reliability (measured by Cronbach’s alpha), were calculated. See the preregistration for more details about the literature review and the evaluation of the quality of measurement.
 This resulted in the selection of variables measured by four different scales, measuring school-related constructs (i.e., *classroom environment* and *academic self-concept*), personality traits (i.e., *neuroticism*, *conscientiousness*, *openness to new experiences*, *extraversion,* and *agreeableness*), life satisfaction (i.e*., satisfaction with family*, *satisfaction with friends*, *satisfaction with school*, *satisfaction with the living environment* and *satisfaction with oneself*) and callous-unemotional traits (i.e., *callous*, *unemotional* and *uncaring*). For a description of all environmental variables that were considered as well as comprehensive results of the literature review and analysis of the psychometric qualities of the scales, see the preregistration of this study.

**B. Details integrated item response theory (IRT) model**
Various IRT models are available. Based on an earlier analysis of a subset of the data (see Schwabe et al., 2023) that assessed which IRT model fitted the data the best, we used the generalized partial credit model (GPCM) here, which is an IRT model that is suitable for polytomous and ordinal item data (Muraki, 1992). In the GPCM, answers to a questionnaire are treated as ordered trait levels and the probability of choosing response category *k* over response category *k-1* for item *l* is modeled using both latent trait value and item parameters. For example, if an ADHD item (like ‘*I am always organized*) is assessed on a scale with 4 answer categories (e.g., “1 – *never*”, “2 – *sometimes*”, 3 – *often* and 4 – *always),* a respondent who answers “*sometimes*”, is considered to have chosen “*sometimes*” over “*never*” and “*often*” over “*sometimes*”, but to not have chosen “*always*” over “*often*”. This can also be interpreted intuitively as if a respondent ‘passes’ through all of the preceding ordered response categories and stops at the final response which is the one that most accurately reflects his or her situation. For each successive response category, the GPCM assumes that the probability of answering category *k* over *k-1* for item l is following a conditional probability dependent on a twin’s latent trait _ij_, the discrimination parameter and thresholds of every item category of item *l*, β_lk_, that the twin has to pass through in order to reach the next category *k*. The discrimination parameter is comparable to a factor-loading in the structural equation modeling (SEM) framework and can be interpreted as the ability of an item to discriminate between the various levels of the latent trait (e.g., the various degrees of inattentiveness). To identify this IRT model, in all estimated models, the mean of the latent trait was set to zero as well as the first threshold of all items (i.e., μ = 0 and β_l1_ = 0).

**C. Details estimation procedure/MCM algorithm**
For this estimation, we used a Markov Chain Monte Carlo (MCMC) algorithm (Gibbs sampling) to approximate the posterior density distribution for the estimated parameters. For the MCMC estimation, we used the freely obtainable MCMC software package JAGS (Plummer, 2003) and for further data handling, the statistical programming language R (R Development Core Team, 2008) was used.

For every estimated model, we used a burn-in phase of 25,000 iterations and a total of 30,000 iterations from two separate Markov chains for estimating the posterior distributions. For all relevant parameters, we calculated posterior point estimates and 95% HPD intervals. To assess the convergence of a model, we used trace plots and calculated the Gelman-Rubin convergence diagnostic for all estimated parameters.

**D. Results of models including also non-significant gene-environment interactions

*Hyperactivity-impulsivity***

*Personality traits*

| **Parameter** | **Point estimate** | **95% HPD** |
| --- | --- | --- |
| $\beta_{0A}$ | 0.16 | [0.11;0.21] |
| $\beta_{0E}$ | 0.23 | [0.18;0.28] |
| *A x Agreeableness* | 0.14 | [0.00;0.28] |
| *A x Conscientiousness* | 0.23 | [0.10;0.35] |
| *A x Openness to new exp.* | -0.02 | [-0.16;0.12] |
| *A x Extraversion* | 0.03 | [-0.10;0.17] |
| *A x Neuroticism* | -0.16 | [-0.29;-0.02] |

**Table A 1**. *Hyperactivity-impulsivity, personality variables*: estimated parameter values.

*School-related measures*

| **Parameter** | **Point estimate** | **95% HPD** |
| --- | --- | --- |
| $\beta_{0A}$ | 0.20 | [0.14;0.26] |
| $\beta_{0E}$ | 0.24 | [0.18;0.29] |
| *A x Academic self-concept* | 0.23 | [0.12;0.34] |
| *A x Classroom environment* | 0.25 | [0.10;0.37] |

**Table A 2**. *Hyperactivity-impulsivity, school-related variables*: estimated parameter values.

*Life satisfaction*

| **Parameter** | **Point estimate** | **95% HPD** |
| --- | --- | --- |
| $\beta_{0A}$ | 0.22 | [0.15;0.28] |
| $\beta_{0E}$ | 0.24 | [0.18;0.30] |
| *A x Family* | 0.18 | [0.05;0.31] |
| *A x Friends* | 0.12 | [-0.01;0.25] |
| *A x School* | 0.10 | [-0.04;0.23] |
| *A x Living environment* | 0.06 | [-0.07;0.18] |
| *A x Self* | 0.17 | [0.04;0.30] |

**Table A 3**. *Hyperactivity-impulsivity, life satisfaction*: estimated parameter values.

*Callous-unemotional traits*

| **Parameter** | **Point estimate** | **95% HPD** |
| --- | --- | --- |
| $\beta_{0A}$ | 0.19 | [0.14;0.25] |
| $\beta_{0E}$ | 0.25 | [0.20;0.31] |
| *A x Callous* | -0.20 | [-0.36;-0.04] |
| *A x Uncaring* | 0.26 | [0.07;0.45] |
| *A x Unemotional* | 0.08 | [-0.08;0.23] |

**Table A 4**. *Hyperactivity-impulsivity, callous-unemotional traits*: estimated parameter values.

**Inattention**

*Personality traits*

| **Parameter** | **Point estimate** | **95% HPD** |
| --- | --- | --- |
| $\beta_{0A}$ | 0.18 | [0.05;0.34] |
| $\beta_{0c}$ | 0.16 | [0.02;0.28] |
| $\beta_{0E}$ | 0.48 | [0.37;0.60] |
| *A x Agreeableness* | 0.03 | [-0.15;0.20] |
| *A x Conscientiousness* | 0.48 | [0.31;0.66] |
| *A x Openness to new exp.* | -0.10 | [-0.28;0.08] |
| *A x Extraversion* | 0.12 | [-0.05;0.30] |
| *A x Neuroticism* | -0.13 | [-0.32;0.04] |

**Table A 5**. *Inattentiveness, personality variables*: estimated parameter values.

*School-related measures*

| **Parameter** | **Point estimate** | **95% HPD** |
| --- | --- | --- |
| $\beta_{0A}$ | 0.27 | [0.12;0.42] |
| $\beta_{0C}$ | 0.10 | [0.0;0.20] |
| $\beta_{0E}$ | 0.51 | [0.39;0.63] |
| *A x Academic self-concept* | 0.43 | [0.29;0.60] |
| *A x Classroom environment* | 0.18 | [-0.03;0.39] |

**Table A 6**. *Inattentiveness, school-related measures*: estimated parameter values.

*Life satisfaction*

| **Parameter** | **Point estimate** | **95% HPD** |
| --- | --- | --- |
| $\beta_{0A}$ | 0.36 | [0.20;0.50] |
| $\beta_{0C}$ | 0.07 | [0.00;0.15] |
| $\beta_{0E}$ | 0.52 | [0.40;0.64] |
| *A x Family* | 0.21 | [0.06;0.36] |
| *A x Friends* | -0.14 | [-0.28;-0.01] |
| *A x School* | 0.28 | [0.12;0.45] |
| *A x Living environment* | 0.11 | [-0.03;0.26] |
| *A x Self* | 0.26 | [0.12;0.41] |

**Table A 7**. *Inattentiveness, life satisfaction*: estimated parameter values.

*Callous-unemotional traits*

| **Parameter** | **Point estimate** | **95% HPD** |
| --- | --- | --- |
| $\beta_{0A}$ | 0.20 | [0.07;0.34] |
| $\beta_{0C}$ | 0.12 | [0.02;0.22] |
| $\beta_{0E}$ | 0.58 | [0.46;0.70] |
| *A x Callous* | -0.66 | [-1.14;-0.34] |
| *A x Uncaring* | 0.34 | [0.05;0.66] |
| *A x Unemotional* | 0.24 | [-0.01;0.49] |

**Table A 8**. *Inattentiveness, callous-unemotional traits*: estimated parameter values.

**D. Estimates of the main effects of environmental measures**Following the procedure described in more detail in van der Sluis et al. (2012), four different parameters were estimated per environmental variable: the effect of the environmental variable on the hyperactivity scores of a twin (e.g.,$\beta_{m}$) and the co-twin of the same family (e.g., $\beta_{mc}$), separately for MZ twin families and DZ families. Displays are presented below for hyperactivity-impulsivity and inattentiveness separately.

***Hyperactivity-impulsivity***

See Table A9 for all estimates, separately per environmental variable. Estimates of the main effects of environmental measures were all non-significant and low (ranging from -0.01 for *classroom* *environment* to 0.10 for *conscientiousness* and *academic* *self-concept*) and very similar across zygosity (e.g., MZ and DZ twins) and within the same family (e.g., twin and co-twin).

| **Variable** | **Academic self-concept** | | **Classroom environment** | | **Conscientiousness** | |
| --- | --- | --- | --- | --- | --- | --- |
|  | **MZ twins** | | **MZ twins** | | **MZ twins** | |
| **Parameter** | $\beta_{m}$ | $\beta_{mc}$ | $\beta_{m}$ | $\beta_{mc}$ | $\beta_{m}$ | $\beta_{mc}$ |
| **Point estimate** | 0.09 | 0.03 | 0.04 | -0.01 | 0.10 | 0.04 |
| **95% HPD** | [0.04;0.13] | [-0.02;0.08] | [-0.01;0.08] | [-0.06;0.05] | [0.06;0.14] | [0.00;0.08] |
|  | **DZ twins** | | **DZ twins** | | **DZ twins** | |
| **Point estimate** | 0.10 | 0.03 | 0.04 | 0.03 | 0.08 | 0.01 |
| **95% HPD** | [0.07;0.14] | [0.00;0.07] | [0.00;0.08] | [-0.01;0.06] | [0.04;0.11] | [-0.04;0.05] |

**Table A9**. *Hyperactivity-impulsivity.* Estimates of main effect of environmental variables, estimated separately for twin and co-twin and MZ and DZ families. $\beta_{m}$ refers to the effect of the environmental variable on the hyperactivity of the twin self, $\beta_{mc}$to the effect of the environmental variable on the hyperactivity scores of the co-twin.

***Inattentiveness***See Table A10 for all estimates, separately per environmental variable. Although the point estimates of the main effects of environmental measures were generally larger than those found for the hyperactivity-impulsivity model, most estimates were not significant (e.g., the 95% HPD interval including zero). The only two environmental measures that showed significant effects on the *inattentiveness* scores of the twin self (e.g., $\beta_{m}$) were the personality trait *conscientiousness* and *academic self-concept*. Interestingly, the estimates were identical for MZ twins and DZ twins, suggesting that there were no differences in effect across zygosity: The point estimate for *conscientiousness* was equal to $\beta_{m}$= 0.15 (95% HPD: [0.10;0.21]) and equal to $\beta_{m}$= 0.16 (95% HPD: [0.10;0.22]) for academic self-concept for both MZ and DZ twins.

| **Variable** | **Satisfaction with school** | | **Satisfaction with oneself** | | **Callous** | | **Conscientiousness** | | **Academic self-concept** | |
| --- | --- | --- | --- | --- | --- | --- | --- | --- | --- | --- |
|  | **MZ twins** | | **MZ twins** | | **MZ twins** | | **MZ twins** | | **MZ twins** | |
| **Parameter** | $\beta_{m}$ | $\beta_{mc}$ | $\beta_{m}$ | $\beta_{mc}$ | $\beta_{m}$ | $\beta_{mc}$ | $\beta_{mc}$ | $\beta_{mc}$ | $\beta_{m}$ | $\beta_{mc}$ |
| **Point estimate** | 0.11 | -0.01 | 0.07 | -0.02 | -0.37 | -0.12 | 0.15 | 0.04 | 0.16 | 0.07 |
| **95% HPD** | [0.04;0.18] | [-0.07;0.06] | [0.01;0.13] | [-0.08;0.04] | [-0.45;-0.30] | [-0.19;0.05] | [0.10;0.21] | [-0.01;0.09] | [0.10;0.22] | [0.01;0.13] |
|  | **DZ twins** | | **DZ twins** | | **DZ twins** | | **DZ twins** | | **DZ twins** | |
| **Point estimate** | 0.11 | 0.02 | 0.07 | 0.07 | -0.37 | -0.10 | 0.15 | -0.03 | 0.16 | 0.02 |
| **95% HPD** | [0.04;0.18] | [-0.04;0.07] | [0.01;0.13] | [0.01;0.12] | [-0.45;-0.30] | [-0.16;-0.05] | [0.10;0.21] | [-0.08;0.02] | [0.10;0.22] | [-0.03;0.07] |

**Table A10**. *Inattentiveness.* Estimates of main effect of environmental variables, estimated separately for twin and co-twin and MZ and DZ families. $\beta_{m}$ refers to the effect of the environmental variable on the hyperactivity of the twin self, $\beta_{mc}$to the effect of the environmental variable on the hyperactivity scores of the co-twin.
